# Back to Basics: Choosing the Appropriate Surface Disinfectant

**DOI:** 10.3390/antibiotics10060613

**Published:** 2021-05-21

**Authors:** Angelica Artasensi, Sarah Mazzotta, Laura Fumagalli

**Affiliations:** Dipartimento di Scienze Farmaceutiche, Università degli Studi di Milano, Via L. Mangiagalli 25, 20133 Milano, Italy; angelica.artasensi@unimi.it (A.A.); sarah.mazzotta@unimi.it (S.M.)

**Keywords:** antimicrobial, disinfectant, surface disinfection, fomite, surface contamination, microorganisms

## Abstract

From viruses to bacteria, our lives are filled with exposure to germs. In built environments, exposure to infectious microorganisms and their byproducts is clearly linked to human health. In the last year, public health emergency surrounding the COVID-19 pandemic stressed the importance of having good biosafety measures and practices. To prevent infection from spreading and to maintain the barrier, disinfection and hygiene habits are crucial, especially when the microorganism can persist and survive on surfaces. Contaminated surfaces are called fomites and on them, microorganisms can survive even for months. As a consequence, fomites serve as a second reservoir and transfer pathogens between hosts. The knowledge of microorganisms, type of surface, and antimicrobial agent is fundamental to develop the best approach to sanitize fomites and to obtain good disinfection levels. Hence, this review has the purpose to briefly describe the organisms, the kind of risk associated with them, and the main classes of antimicrobials for surfaces, to help choose the right approach to prevent exposure to pathogens.

## 1. Introduction

**Figure 1:**
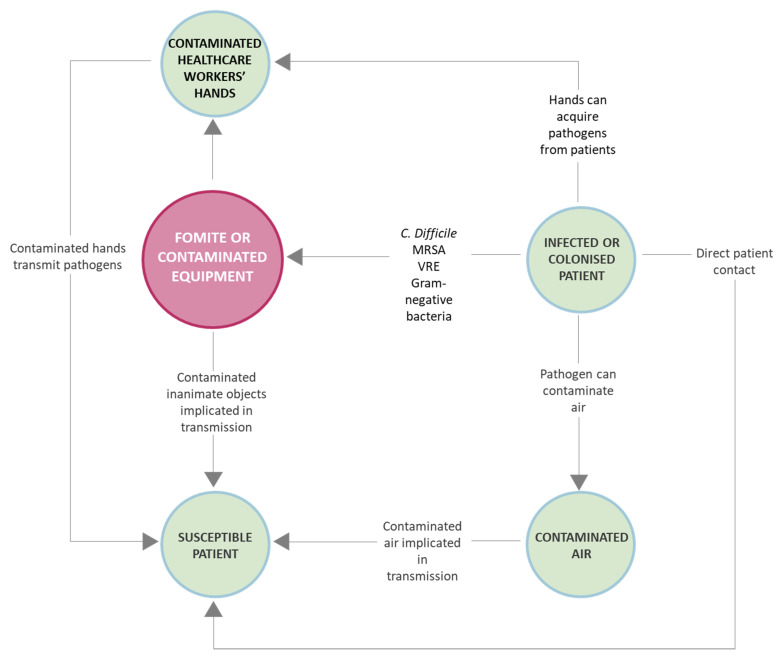
Generic transmission route.

In built environment, especially considering an indoor lifestyle, touching objects or surfaces which surround us is integral to everyday life. Such objects or surfaces if contaminated are called fomites and, in the 21st century, their role in disease transfer is higher than ever in human history. Indeed, most microorganisms found in the indoor environment are inactive, dormant, or dead and either show no impact on human health or are even beneficial. Nevertheless, fomites can become contaminated by pathogenic organisms which have a variety of negative health consequences. In fact, microorganisms can survive even for many months and multiply on surfaces or objects [1], leading to the development of secondary reservoirs. As a consequence fomites can serve as a mechanism for transfer between hosts, just think to doorknobs, elevator buttons, handrails, phones, keyboards, writing implement, etc., that are touched by a person that afterward will handle other objects (Figure 1).

Furthermore, experimental data show that touching a fomite carries approximately the same risk for the acquisition of a lot of microorganisms (i.e., Methicillin-Resistant *Staphylococcus aureus*—MRSA, Vancomycin-Resistant *Enterococcus*—VRE, and *Clostridium difficile*) on hands as touching an infected patient [2,3,4,5]. Consequently, preventing transmission of pathogens with disinfection procedures must be carried out not only in the high-risk sectors, like laboratories, operating rooms, intensive care units, or food-handling settings but also for hygienic behavior in everyday life on floors and on all the surfaces that frequently are touched with hands.

Therefore, environmental disinfection, hygiene habits, and the consequent maintenance of barriers are crucial in preventing infection from spreading. To develop effective policies and regulations to minimize the risk of transmission is strictly necessary to evaluate which organisms are present on the fomites. Furthermore, the choice of the effective antimicrobial agent is also based on the risk assessment of the microorganisms and the type of fomites 

Public health emergency surrounding the COVID-19 pandemic, stressed the importance of having good biosafety measures and practices, as never before. On these bases, this review has the purpose to briefly describe the organisms, the kind of risk associated with them, and the major characteristic of the main classes of antimicrobials for surfaces to help in choosing the right approach to prevent exposure to pathogens.

## 2. Most Common Microorganisms on Fomites and Associated Risks

The primary goal of disinfecting procedures is the inactivation of organisms on fomites. Generally, microorganisms belong to a diverse group such as bacteria, viral, and protozoan species [6]. These biological agents are widely found in the natural environment and, as a result, they can be found either in many work sectors or household contexts. The majority of these microorganisms are harmless; however, some of them or their metabolites may cause diseases. For example, the transmission of norovirus that causes nonbacterial gastroenteritis outbreaks is fomite-mediated as well as coccidioidomycosis. Furthermore, some of the greatest concerns regarding antibiotic-resistant bacteria transmission occur via fomite as reported by Julian et al. [7] for *Staphylococcus pseudintermedius*. Therefore, the knowledge of these organisms and their survival is fundamental to choose the right antimicrobial agents and implementing effective tactics.

### 2.1. Bacteria 

**Figure 2:**
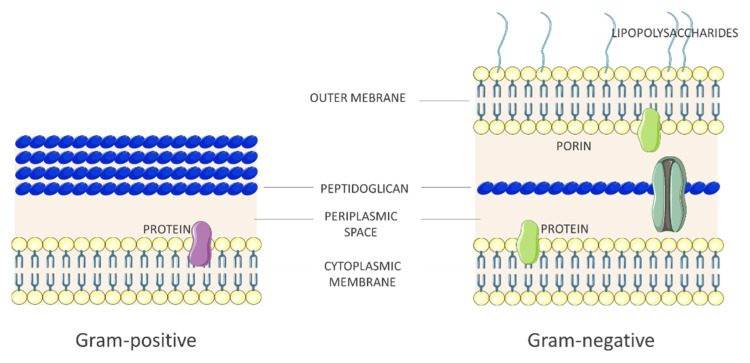
Gram-negative versus Gram-positive cell walls.

Bacteria are single-celled organisms (0.3–1.5 µm) with independent life and replication cycle. Bacterial cells are generally surrounded by two concentric protective layers: an inner cell membrane and an outer cell wall [8]. The cytoplasmatic membrane shares a similar structure to the eukaryote’s one, but there are no sterols. Here, proteins involved in the energy production can be found like some respiratory chain protein as well as photosynthetic protein in photosynthetic bacteria that lack chloroplast. Among the proteins that constitute the cell wall, the main one is peptidoglycan (PGN), also known as murein, which provides rigidity to the structure and counteracts the osmotic pressure of the cytoplasm. PGN is characterized by a glucidic backbone of alternating units of two azotated carbohydrates, namely N-acetylglucosamine (GlcNAc) and N-acetylmuramic acid (MurNAc). Each MurNAc is cross-linked to a short amino acid chain, which can vary with different bacterial species [9]. The differences in structural characterization of peptidoglycan define two morphological categories: Gram-positive and Gram-negative bacteria (Figure 2).

In Gram-positive bacteria, peptidoglycans make up about 20% of the cell wall dry weight; while in Gram-negative bacteria the thicker peptidoglycan layer contains about 10% of the cell wall dry weight [10]. Furthermore, Gram-positive cell wall has a significant amount (up to 50%) of teichoic and teichuronic acid, which are involved in pathogenesis and play key roles in antibiotic resistance [11].

Certain bacteria may even have a third outermost protective layer called a capsule. Whip-like extensions often cover the surfaces of bacteria—long ones called flagella and short ones called pili—to become motile and seek out nutrients [12]. An alternative resource exploited by some bacteria is the formation of endospores that are dormant and highly resistant cells able to preserve the genetic material. This ruse helps the bacteria to survive even without nutrients or under extreme stress [13].

Among endospore-producing bacteria, the most common are the *Bacillus* and *Clostridium* genera [14]. Table 1 reports several endospore-forming bacteria and their relative clinical manifestations.

Another bacteria’s survival mechanism is the formation of biofilm: clusters of bacteria that are attached to a surface and/or to each other. During biofilm development, bacteria secrete extracellular polymeric substances (EPS) which are crucial to the production of an extracellular matrix [15]. This network maintains cohesion between cells and the surface and protects the accumulation of microorganisms against chemical, biological, and mechanical stressors. In this complex arrangement of cells, there are interstitial void spaces in which water flows so nutrients and oxygen diffuse [16]. As biofilm protects from harsh conditions and resistance towards antibiotics, it represents a serious global health concern. Furthermore, biofilm is involved in persistent chronic infections [17,18] and may potentially contribute to their pathogenesis [19]. In addition, some bacteria can produce a polysaccharide exocellular slime (the glycocalyx), which adheres to compromised tissue or the surfaces of biomaterials [20]. In fact, the glycocalyx is a fundamental factor in the persistence of infection linked to the prosthetic device.

### 2.2. Virus

**Figure 3:**
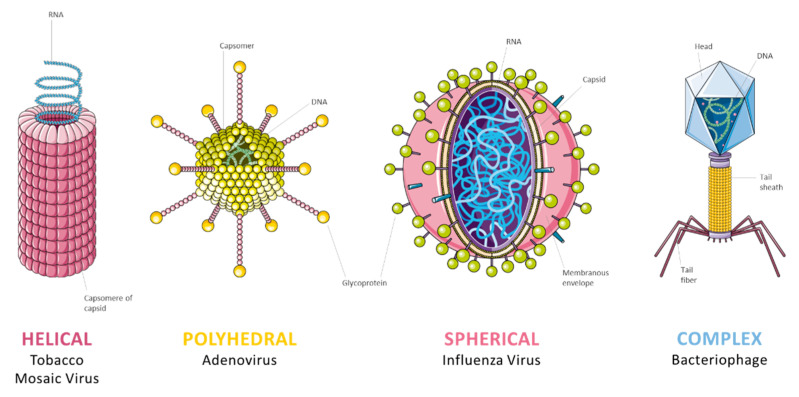
Types of viruses architecture.

Viruses are subcellular organisms with submicroscopic dimensions (nm). Their core has either DNA (deoxyribonucleic acid) or RNA (ribonucleic acid) as genetic material. The core is covered by a protein coat [21], called the capsid, whose role is to protect it from degradation. Furthermore, the protein coat allows the virus to attach to a specific receptor of the host cell. In fact, viruses are obligate intracellular parasites [22], so they need host ribosomes to synthesize viral proteins. Capsid proteins are codified by the viral genome, whose short length entails a limited number of proteins with a specific function. This leads to a capsid constituted by repetitive units of one or a few proteins combined in a continuous structure [23], which can have a helicoidal or geometric symmetry. The former is characterized by a helicoidal distribution around the nucleic acid while the latter by a polyhedral or a spherical shape. Besides these styles, a few viruses have a complex architecture like poxviruses, geminiviruses, and many bacteriophages [24] (Figure 3).

Furthermore, some viruses show a further shell, called envelope, constituted by viral proteins and lipids. The envelope shields the virus from the immune system’s detection and, in addition, facilitates the fusion with the host cell membrane [23].

### 2.3. Fungi

**Figure 4:**
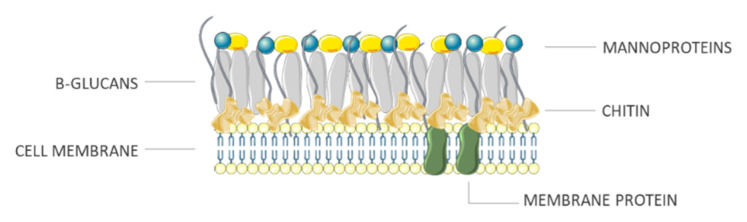
Fungal wall.

Fungi are a large group of eukaryotic organisms, mono or pluricellular, that also include yeast and molds. As these organisms have a rigid cell wall (rich in chitin and other polysaccharides, especially glucans as depicted in Figure 4) [25], they feed themselves secreting digestive enzymes and by absorbing organic matter from the environment: thus, they are called heterotrophic organisms. Some fungi can live by decomposing dead organic matter (saprobic) while others are a parasite of organisms, even fungi, or have developed complex symbionts as in lichens and mycorrhizae [26].

### 2.4. Microbiological Risk Assessment

According to the Code of Practice to the Safety, Health and Welfare at Work (Biological Agents) Regulation 2020 [27] the biological agents can be classified into four risk groups, reported in Table 2. The classification takes into account:

- Virulence—Ability of the microorganism to penetrate and multiplicate inside the host organism;
- Pathogenicity—Severity of the disease that may result;
- Transmissibility—Capability of the microorganism to be transmitted from one organism to another;
- Treatment—Availability, if any, of effective prophylaxis or therapy.

Disinfection policies should be also based on risk assessment to control cross-contamination while reducing the risk caused by exposure to infectious agents. The evaluation of the surface’s risks and type together with the nature of the pathogen agent(s) should lead to the use of an appropriate and effective antimicrobial agent. Such approaches must be learned by everyone since their implementation in the routine measure improves both cleaning performance and infection prevention [28].

However, as far as possible, the number of antimicrobials to be used should be limited not only for healthy and economic reasons but also to reduce environmental pollution. Not least, the discharge of waste biocides into the environment may promote the development of both biocide and antibiotic resistance [29].

## 3. Factors That Affect the Activity of Antimicrobials

The activity of the antimicrobial agents depends on several factors, some of which are intrinsic qualities of the organism, others derived from the chemicals and external physical environment. More specifically need to be listed:

- Number and type of microorganism
  No disinfectant can effectively act on all microorganism classes. So proper choice of chemical germicides is fundamental. Furthermore, some microbes can persist on surfaces showing resistance to these products: for example, the production of endospores or biofilm matrix protects the pathogens from environmental influences [13,30].
- Type and concentration of the antimicrobial
  After choosing the proper disinfectant, the concentration of the active ingredient is a key factor: the influence of changing in the concentration of the active(s) can be measured experimentally, with the determination of the kinetics of inactivation. Moreover, the knowledge of the effect of dilution or concentration on the activity of a sanitizing agent provides some valuable information that could lead to a reduction in the exposure time.
  Furthermore, microbicidal concentration is also a central concept in the microbial resistance field and it is especially important nowadays with increasing knowledge and restrictions on the environmental discharges of potentially harmful chemicals [31].
- pH of the solution
  The pH of the solution can affect the efficacy of the disinfection in two ways: a change in the agent itself and a change in the interactions between the microbicide and the microbial cell.
  For example, several microbicides are effective in their unionized form (Table 3). Thus, the pH level would affect their degree of dissociation and would decrease their overall activity. In contrast, other molecules are more effective in their ionized form. Besides these considerations, it should also be kept in mind that any alteration of the pH level could affect the compound’s stability.
  As a matter of fact, disinfectant products in the sanitary field are formulated to guarantee, at a certain level of pH, maximum germicidal efficacy.

- Formulation
  The formulation of a disinfectant deeply affects its activity. Several excipients, such as solvents, surfactants, thickeners, chelating agents, colors, and fragrances, can be found in these products; they can interact with the microorganisms or with the active itself and ultimately affect the activity of the formulated product. Most of the information on the effect of different excipients on the activity of disinfectants are not available, since they are often trade secrets.
- Length of exposure
  The microbicidal activity of chemicals usually increases with the rise of contact time. However, there is not a direct correlation between contact time and microbicidal activity, maybe due to other factors. Contact times for disinfectants are specific for each material and manufacturer. Therefore, all recommendations for use of disinfectants should follow manufacturers’ specifications that must be reported on the label.
- Temperature
  Temperature can be an important parameter that influences the pathogen’s survival. High temperature can impact vital proteins and enzymes, as well as the genome. Moreover, high temperature can boost and speed up the germicidal activity of many chemicals resulting in reduced time and improved efficacy. As a drawback, high temperature can accelerate the evaporation of the chemicals and also degrade them. Particular care is needed in using and in stocking such chemicals in tropical regions, where their shelf-life may be reduced because of high room temperature;
- Type of surfaces and precleaning process
  The location of microorganisms must be considered as well: to sanitize an instrument with multiple pieces or joints and channels is more difficult than a flat surface. Only surfaces that directly contact the germicide will be sanitized. Indeed, the presence of dirt is the principal reason for disinfection failure, since it could interact with the microbicide, reducing its availability or interact with the microorganisms, giving protection. Moreover, material characteristics of the surface may influence the survival of microorganisms as well: for example, porous surfaces are more difficult to clean and, consequently, to disinfect. Pretreatment of surfaces, especially when visibly soiled, is fundamental to ensure or improve the microbicidal efficacy of the disinfection procedure.

Besides the activity that is influenced by the factors listed upon, ideally, an antimicrobial agent should: (1) have a wide spectrum against microorganisms; (2) be rapid in its action; (3) be compatible with many materials; (4) be safe for humans and the environment.

## 4. Most Common Antimicrobial Classes

**Figure 5:**
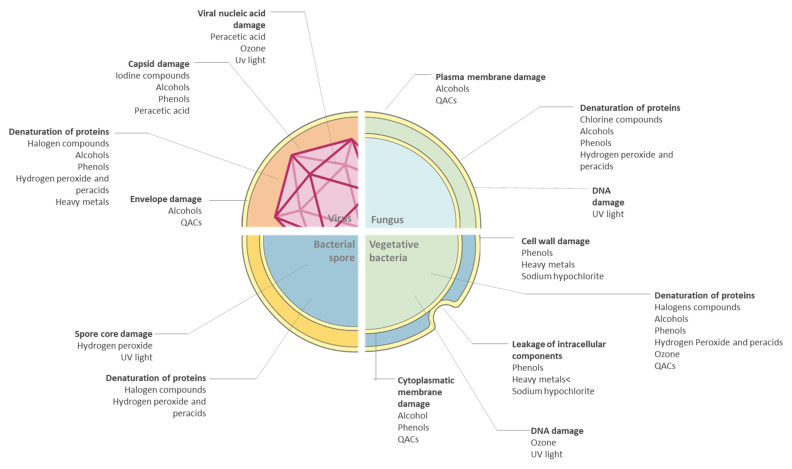
Mechanisms of biocide actions on microorganisms.

At present, there are numerous substances to be used on surfaces that are claimed as antimicrobial agents and they are formulated alone or in combination. The most common disinfectants can be roughly divided as halogens, alcohols, quaternary ammonium compounds (QACs), peroxigens, ozone, and UV. Generally, these antimicrobials damage a specific part of the microorganism as reported in Figure 5.

### 4.1. Halogens

#### 4.1.1. Chlorine Compounds

Historically, the most widely used antimicrobial agents belonging to halogens are chlorine and chlorine releasing compounds.

Since elemental chlorine gas (Cl_2_) is hazardous it must be banned either from workplaces or household environment and substituted by chlorine-releasing agents.

The most commonly used chlorine-releasing agent is sodium hypochlorite (NaOCl), universally known as bleach, which is characterized by high microbicidal efficacy, low toxicity to humans, and low cost, but suffers the disadvantages of being irritant and corrosive. Nevertheless, ceramics, methylacrylate, or cement are not sensitive to bleach. More specifically, sodium hypochlorite is potentially bactericidal, virucidal, fungicidal, mycobactericidal, sporicidal. Hence it plays an important role in the surface disinfection of healthcare facilities and medical equipment.

The concentration of sodium hypochlorite sold for domestic purposes is around 5–6%, with a pH around 11 and it is irritant; while in higher concentration, 10–15%, with a pH around 13, it burns and it is corrosive. According to the Laboratory biosafety manual [32] published by the World Health Organisation (WHO): “*A general all-purpose laboratory disinfectant should have a concentration of 1 g/L available chlorine. A stronger solution, containing 5 g/L available chlorine, is recommended for dealing with biohazardous spillage and in the presence of large amounts of organic matter. Sodium hypochlorite solutions, as domestic bleach, contain 50 g/L available chlorine and should therefore be diluted 1:50 or 1:10 to obtain final concentrations of 1 g/L and 5 g/L, respectively. […] Surfaces can be decontaminated using a solution of sodium hypochlorite (NaOCl); a solution containing 1 g/L available chlorine may be suitable for general environmental sanitation, but stronger solutions (5 g/L) are recommended when dealing with high-risk situations*.”

Once sodium hypochlorite dissolves in water (Equations (1)–(3)) the two compounds that cause disinfection via oxidation are generated, namely hypochlorite ion (OCl^−^), a weak base, and its corresponding acid, hypochlorous acid (HOCl), whose percentage is determined by water’s pH and which is the most active between the two [33,34]. In fact, hypochlorous acid, due to no electronic charge, better penetrate the microorganism cell wall or any protective layer and effectively kills them by oxidating the side chains of proteins’ amino acids [35,36].

(1) $$OCl^{-}+H_{2}O⇆HOCl+OH^{-}$$

(2) $$HOCl+H^{+}+Cl^{-}\;⇆Cl_{2}+H_{2}O$$

(3) $$2HOCl+OCl^{-}\;\to ClO_{3}^{-}+2Cl^{-}+H^{+}$$

It is also common to express the concentration of chlorine compounds in terms of available chlorine or free available chlorine (FAC). The term FAC refers to the mixture of oxidizing chlorine forms that have a chlorine atom in the 0 or −1 oxidation state and are not combined with ammonia or organic nitrogen.

Sodium hypochlorite is characterized by high instability, therefore the FAC value is not so significant: 0.75 grams of activated chlorine evaporate per day. This happens not only when sodium hypochlorite gets heated up, but also when gets in touch with acids, sunlight, specific metals, toxic and corrosive gases, included chlorine itself [37,38]. 

Sodium hypochlorite solution is an inflammable weak base and these characteristics must be considered during its use and storage. Because of these reasons, formulation and conditions for the application should minimize the formation of by-products and even chloramines [39]. The overall stoichiometry of degradation is shown in Equation 3.

**Figure 6:**
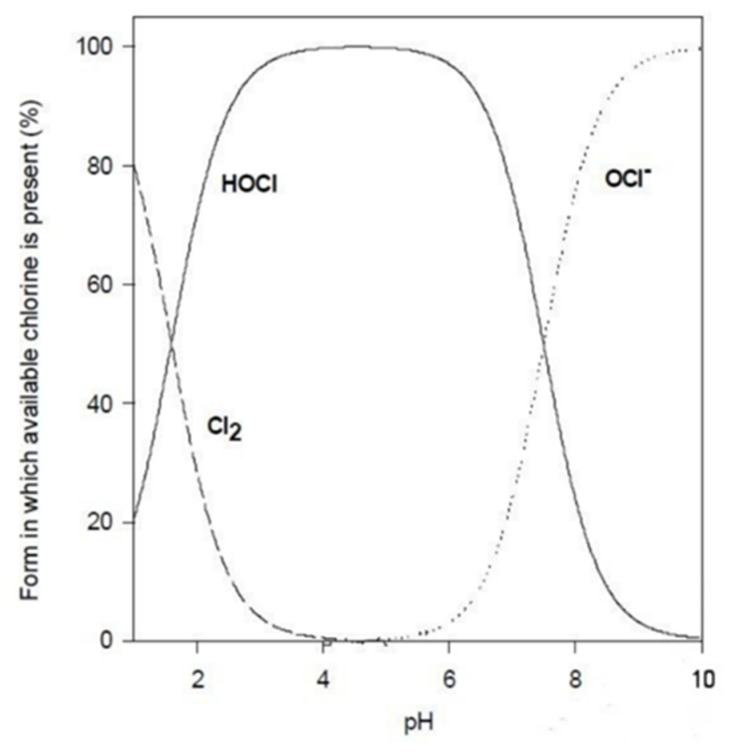
Active chlorine species concentration at different pH values [42].

Thus disinfection’s efficacy of chlorine releasing agents depends on the water’s pH and FAC. Chlorine disinfection against vegetative bacteria, fungi, and yeast, as well as fungal conidia and viruses, is preferable at alkaline NaOCl solutions; although the germicidal efficacy is even greater when pH value is around 5.5 and 8 [39,40]. Furthermore, Kuroiwa et al. [41] proved that adjusting the pH around 5 by weak acidification with acetic acid, resulted in a shortened killing time of all the *B. subtilis* JCM1465 spores by one-third. On the contrary, this preparation killed all of the non-spore-forming bacteria within 30 seconds as quickly as NaClO solution without acidification.The importance of the pH level is shown in Figure 6. At a pH of 7, the concentration of hypochlorous acid is 80%, while when the pH value is around 8, the concentration drops to 20%.

The pH value of the solution is fundamental either for the bactericidal activity or for the shelf life: at 25–35 °C, neutralized-NaOCl solutions (pH 7) expires in a few hours, generated NaOCl (gNaOCl) solutions (produced by electrolysis of a salt (NaCl solution, pH 9) last 6 days, while stabilized NaOCl solutions (pH 9–11) persist more than 30 days [43].

Sodium hypochlorite is widely used, not only as a surface antimicrobial but also in water treatment, water disinfection, and bleaching in the textile industry. Furthermore, it can be used to avoid crustaceans and algae formation in cooling towers.

As an alternative, calcium hypoclorite (Ca(OCl) _2_) also known as HTH (high test hypochlorite) can be used as well. HTH is sold in granular form that, once in solution, achieves a pH of 9–11 and it is as stable as NaOCl [43].

**Figure 7:**
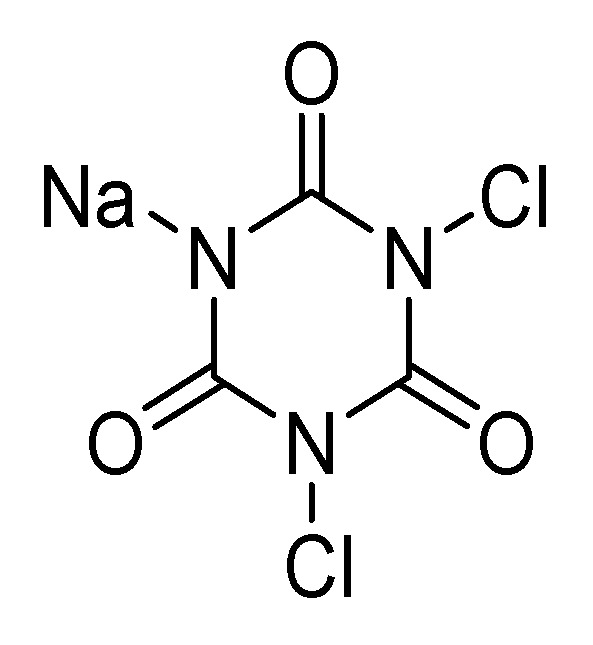
Structure of sodium dichloroisocyanurate (NaDCC).

Another chlorine releasing agent that has been explored as an alternative to sodium, or calcium, hypochlorite is sodium dichloroisocyanurate (NaDCC). This compound is the sodium salt of a chlorinated hydroxytriazine (Figure 7).

This disinfectant is available as a stable powder that produces solutions that have a pH level of around 6 and expire within hours [43]. These solutions are more susceptible to inactivation by organic matter than NaOCl [44,45,46]. NaDCC is often used as a broad-spectrum disinfectant since it has been reported to generally achieve similar disinfection activities to chlorine, while results to be less corrosive. On stainless steel, Bloomfield et al. [47] reported lower ME (microbiocidal effect) values following a 5-min exposure to 250 ppm NaDCC compared to NaOCl at the same concentration against *S. aureus* (2.4 vs. 4.9 to <6.2 log reduction), *Pseudomonas aeruginosa* (3.7 vs. 3.7–4.3 log reduction), and *Enterococcus faecium* (2.2 vs. 3.1 log reduction). At 2500 ppm, both NaDCC and NaOCl achieved at least a 6 log reduction in each tested organism. Gallandat et al. [48] observed similar efficacies of NaOCl, gNaOCl, NaDCC, and HTH (5000 ppm) against both *E. coli* and *Pseudomonas* phage Phi6 after 10–15 min on several nonporous surfaces, with minimum 5.9 and 3.1 log reductions, respectively. At higher concentrations, Aarnisalo et al. [49] observed 3.1 and 0.5 log reductions (without/with 2% pork meat) in *Listeria monocytogene* after 30 seconds exposure to 0.04%(*w/v*) NaDCC and >3.6 and 0.3 log reductions (without/with 2% pork meat) after 30 seconds exposure to 0.2% (*w/v*) NaOCl. Interestingly, the entry containing hypochlorite as an antibacterial agent and anionactive tensides as cleaning compounds were considered to be much more efficient (3.8 and 2.2 log reductions, without/with 2% pork meat) than the hypochlorite disinfectant, probably due to the inactivation of the NaOCl by the organic matter. 

To be effective against bacteria and spores, an adequate concentration of HOCl is required; in Table 4 are reported the recommended dilutions of each chlorine releasing compound mentioned until now to significantly reduce the risk of transmission. The surface conditions, the main advantages, and drawbacks have also been considered. 

#### 4.1.2. Iodine Compounds

Although less reactive than chlorine, iodine solution has a broad spectrum of antimicrobial activity against both gram-negative and gram-positive bacteria, fungi, protozoa, and even bacterial spores [12], while it is not so effective as virucidal [50]. Many investigations identified elemental iodine I_2_ and hypoiodous acid (HIO) as the two most powerful antimicrobials agents among the several iodine species.

(4) $$I_{2}+H_{2}O⇆HIO+I^{-}+H^{+}$$

(5) $$HIO⇆IO^{-}+H^{+}$$

(6) $$3HIO+3OH^{-}⇆IO_{3}^{-}+2I^{-}+3H_{2}O$$

The dissociation constant of hypoiodous acid is 4.5 × 10^−13^ and it reveals that the formation of hypoiodite ion (IO^−^) in an aqueous solution is insignificant. The percentages of the species (see Equations (4)–(6)) are directly related to the pH level of the solution and, to a much lesser extent, to the temperature.

**Figure 8:**
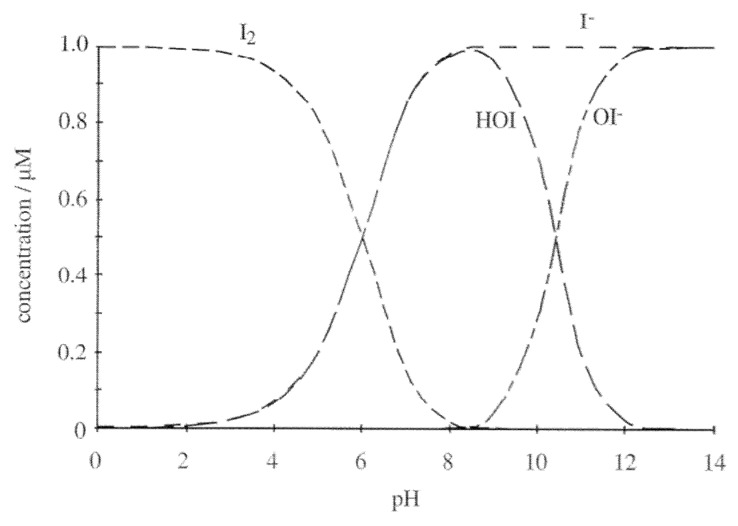
pH-dependent speciation of iodine [51].

Figure 8 shows I_2_ hydrolysis data at different pH values and it is clear that the highest concentrations of the antimicrobial species are present in the acid range. In fact, when the solution is alkaline, several iodine species that have no apparent antimicrobial activity can also be generated. Iodate formation could not be a problem if the pH value stays below 8 and the contact time of disinfection is accomplished in the first 30 min.

Historically iodine solutions or tinctures have been primarily used by health professionals as antiseptics on skin or tissue. Unfortunately, aqueous solutions are generally unstable so a combination of iodine and a solubilizing agent or carrier has been formulated. These combinations, called iodophor, have been used both as antiseptics and disinfectants, retaining the germicidal efficacy of iodine but being more stable and relatively free of toxicity and irritancy [39]. They have been developed to slowly release iodine (I_2_) from the complex, which can be a cationic surfactant, non-ionic, polyoxymer, or polyvinylpyrrolidone [52]

**Figure 9:**
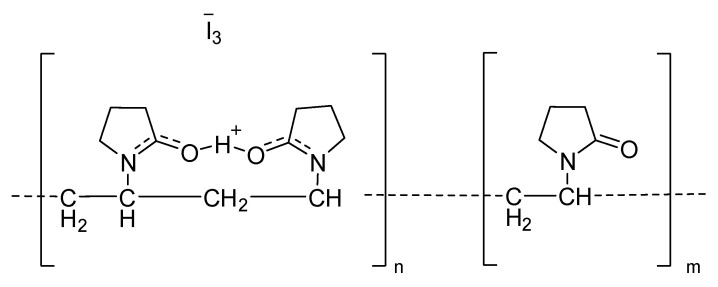
Structure of povidone-iodine complex.

The most known and widely used iodophor is povidone-iodine, Figure 9. Regarding this complex, Block et al. observed 3.14, 3.49, 3.47, and 3.78 log reduction, after 1.5 min for VRE, *E. faecalis,* and methicillin-resistant and methicillin-sensitive *S. aureus*, respectively [53].

Surfactant iodophor, when used, may add a further detergency activity, even though iodine is chemically less reactive than chlorine. Moreover, surfactant iodophor is less affected by the presence of organic matter than chlorine.

An iodophor, when used at 25 ppm (parts per million of available iodine), is considered to act as a sanitizer, however, when the same product is applied at 75 ppm falls into the disinfectant category.

After its release, iodine can quickly penetrate the cell wall of a microorganism and oxidize thiol groups leading to disruption of proteins and nucleic acids structures [39].

### 4.2. Alcohols

#### 4.2.1. Alifatic Alcohols

**Figure 10:**
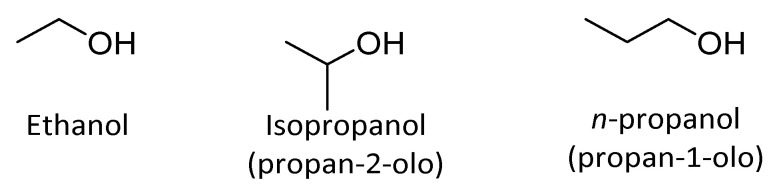
Antimicrobial alcohols.

Among the several aliphatic alcohols that exhibit microbicidal properties ethyl alcohol (ethanol), isopropyl alcohol (isopropanol, propan-2-ol), and *n*-propanol are the most commonly used (Figure 10).

These disinfectants are rapid bactericidal rather than bacteriostatic against vegetative bacteria, included mycobacteria but have no effect on spores. The bactericidal properties of ethanol were examined against several microorganisms for different ranges of time [54]: *P. aeruginosa, Serratia marcescens*, *E. coli,* and *Salmonella typhy* were killed in 10 s by all concentrations of ethanol from 40% to 100% (30% for the *E.coli* entry). *S. aureus* and *Streptococcus pyogenes* were slightly more resistant, being killed in 10 s with concentrations of 60%–95%. Isopropyl alcohol resulted slightly more bactericidal than ethyl alcohol for *E. coli* and *S. aureus* [55]. Furthermore, this category of biocides shows limited fungicidal and virucidal activity especially on lipophilic viruses such as herpes virus, influenza virus, and hepatitis B and C viruses [56,57]. Literature data demonstrate that isopropyl alcohol shows its antimicrobial activity against lipid viruses but it is not active against the nonlipid enteroviruses [58]

These alcohols exert their antimicrobial activity by causing protein denaturation [59,60]. Water plays an important role in the formulation of alcoholic disinfectants because, in its absence, proteins are not readily denatured by alcohol. Therefore a 70% solution of alcohol is a much more effective sanitizer than the pure (99%) product [61], but when the concentration drops below 50% there is no practical value [62]. Concentration can be expressed both by weight/weight percentage (%*w/w*) and, most frequently, by volume/volume percentage (%*v/v*). This value is important since it is linked to the evaporation rate: a higher concentration of alcohol evaporates quickly. The evaporation speed could be an issue if a longer contact time is requested, but the addition of surfactants [63], or combination with alkali, mineral acids, and hydrogen peroxide could overcome this problem [12,24].

Alcohols are fast-acting, easy to use but are not free from limitations that are due to poor detergent properties, toxicity, and, of course, their flammability, which is a big concern. The minimum temperature at which vapors above a volatile combustible substance ignite in air when exposed to flame defines the flashpoint. The higher the concentration, the lower the flashpoint. For example, the flashpoints of 70% ethyl and 70% isopropyl alcohol are 20.5 °C and 21.0 °C, respectively, while the flashpoint of 30% ethyl alcohol is 29 °C [64]. Moreover, even if alcoholic disinfectants are neither corrosive nor staining, they could damage some instruments, by swelling or hardening rubber.

#### 4.2.2. Aromatic Alcohols

Besides aliphatic alcohols, also aromatic ones exhibit antimicrobial properties being effective in sanitization and disinfection, even in the presence of biological fluids. Phenols are the reference standard for the Rideal–Walker (RW) and Chick–Martin tests for disinfectant evaluation [65].

Phenol (C_6_H_5_OH) is an organic compound that consists of a benzene ring bearing a single hydroxy substituent. It appears as a white crystalline solid, which is partially water-soluble (1 g/15 mL water) [66] and it has a pK_a_ value of 10, which means it is classified as a weak acid.

Phenol exerts its antimicrobial activity against vegetative bacteria, both Gram-positive and negative, fungi and viruses but it is not so effective as sporicidal and against acid-fast bacteria.

The biological activity is related to the undissociated molecule, which induces progressive leakage of essential metabolites, including the release of K^+^ [67], leading to membrane damage and consequentially cell lysis, while acting like a protoplasmic poison causing coagulation of the cytoplasm [68].

**Figure 11:**
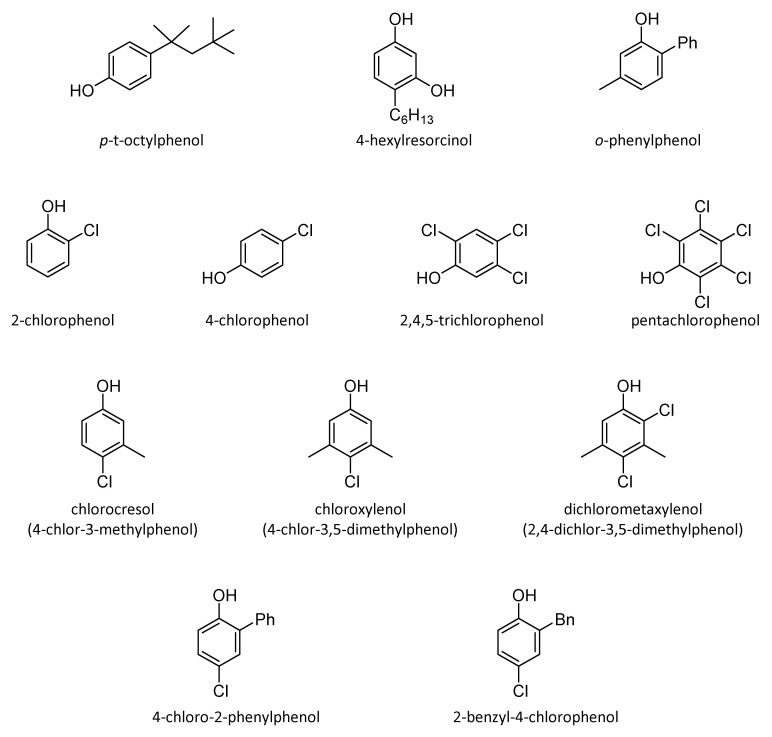
Several microbiocidal phenols.

Phenol is the parent compound but the chemical structure can be modified by replacing one of the hydrogens on the aromatic ring with a different functional group (halogen, alkyl, phenyl, benzyl, etc.). Figure 11 represents several microbicidal phenols.

The structure-activity relationship in the phenol series was investigated by Suter [69]. Regarding the results, it is interesting to notice that the microbiocidal activity increases in derivatives with alkyl chain in the para position, constituted by a maximum of six carbon atoms, since for longer chain the activity drops probably due to the decrease of water solubility. Nitrophenols were evaluated as well; unfortunately, the toxicity increased towards both bacteria and humans and there is also a trend to be inactivated by organic matter. Finally, bisphenolic compounds show activity if they are connected by a methyl linker, sulfur, or oxygen atom, and even if they are directly linked. Augmentation of the efficacy can also be achieved by halogen substitutions.

Among all the derivatives, *o*-phenylphenol and 2-benzyl-4-chlorophenol are widely used as healthcare disinfectants.

As disclosed by published reports, commonly used phenolic compounds show, at their use dilution, antimicrobial efficacy against bacteria, fungi, viruses, including HIV [70,71,72,73]. However, literature reports also that the phenolic disinfectants ‘Stericol’ and ‘Lysol’ show a limited effect on Coxsackie B4, Enterovirus 11, and Poliovirus [74].

Phenols react with certain types of plastic surfaces and are adsorbed by porous material. If not rinsed thoroughly with water, the alcohol residue can cause skin irritation or depigmentation [75]. Moreover, another disadvantage is that phenols are quite expensive, and literature reports demonstrated that they are associated with idiopathic neonatal hyperbilirubinemia in infants [76,77].

### 4.3. Quaternary Ammonium Compounds (QACs)

**Figure 12:**
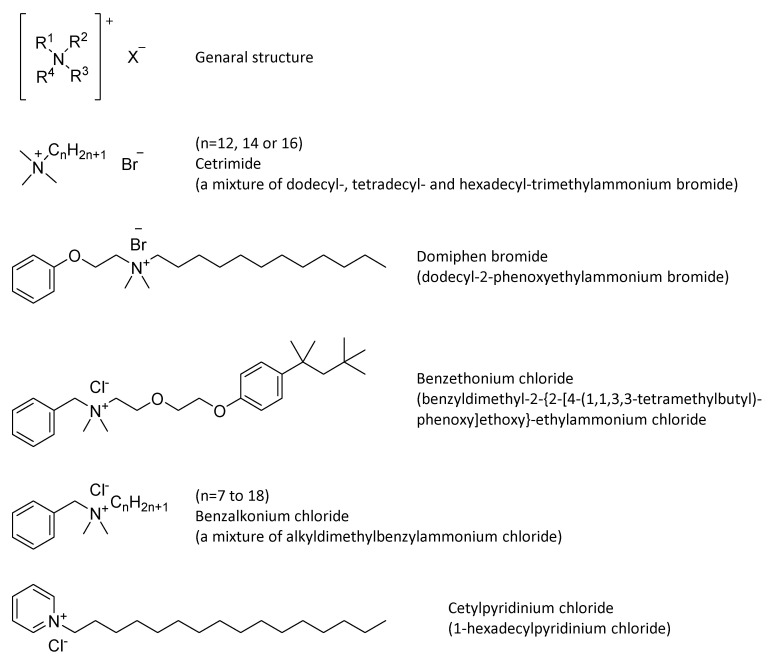
General structure and common QACs.

Quaternary ammonium compounds (QACs) may be considered as amphiphilic substituted compounds, which carry a permanent positively charged nitrogen, counterbalanced by a halide or sulfate moiety. QACs are classified according to the nitrogen substituents, which can include either the type of the carbon chains or the presence of aromatic moieties (Figure 12). The numerous investigations on these chemical structures have increased efficacy while reducing costs.

Demand for these disinfectant agents has increased over the decades, furthermore, their use is not only limited as a germicidal, but they have been widely used also in a variety of industrial, agricultural, clinical applications, and consumer products [78,79,80,81].

Their microbicidal activity is due to their adsorption on proteins or acidic phospholipids in the membrane that leads to the formation of hydrophilic voids. The denaturation of essential cell protein causes cytoplasmic membrane permeability and eventually leads to cell disruption [82]. QACs seems also to be involved in the inactivation of energy-producing enzymes, furthermore, they can bind to DNA [83]. 

Their hydrophobic activity makes them more effective against lipophilic microorganisms. Therefore QACs are solid bactericidal agents, especially against Gram-positive bacteria, and virucidal against enveloped viruses (e.g., herpes simplex, adenovirus, vaccinia) whilst they are not sporicidal and generally not tuberculocidal or virucidal against hydrophilic viruses [84].

QACs are commonly used in ordinary environmental sanitation of noncritical surfaces, such as floors, furniture, and walls. Scientific literature reports that quaternary ammonium-based disinfectants are effective in removing and/or inactivating *S. aureus* and *P. aeruginosa* from computer keyboards, while are not so active against VRE species [85]. Moreover, a recent work by Brown et al. [86] demonstrated that the microbial reduction due to QAC’s activity on glass continues after contact and wetness time.

However, it is important to point up that the efficacy is influenced not only by the compound and surface combinations but even by the product formulation and the water hardness [87]. Indeed, anionic surfactants and high mineral content could lead to insoluble precipitates. Therefore, QAC’s formulation is restricted to nonionic or zwitterionic surfactants, which typically are less effective as cleaning ingredients. Furthermore, some materials, like cellulose-based wipers and gauze pads, absorb these actives, lowering the microbiocidal efficacy [88]. On the other hand, QACs have many advantages like high stability, low color, odorless, and relatively low toxicity (unlike phenols and chlorine bleach). Nevertheless, spraying or fumigation of this chemical disinfectant is not recommended because a few cases disclose occupational asthma as a result of exposure [89,90,91]. When used, these disinfectant agents are often applied with a cloth or wipe that has been soaked in disinfectant, which may contain mixtures of QACs. Benzalkonium chloride (BAC) is one of the most extensively applied QACs, especially in surface disinfection [92]. BAC’s concentration is usually between 0.01 and 1% but can rise at 15% [93]. Other QACs found in disinfection products have similar concentrations. 

### 4.4. Hydrogen Peroxide and Peracids 

**Figure 13:**
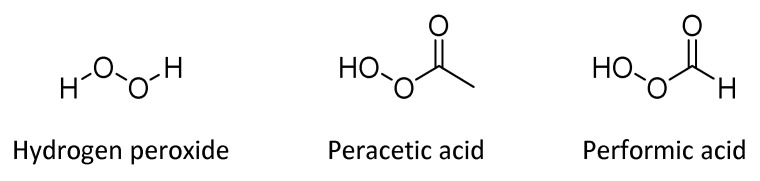
Structures of biocides peroxigen compounds.

Over the years, hydrogen peroxide (H_2_O_2_, HP), represented in Figure 13, has extensively been recognized to have antimicrobial properties against a wide variety of microorganisms, such as bacteria, viruses, spores, and fungi [94,95]. The mechanism involved in the antibacterial effect of HP ascribes to the release of oxygen free radicals (hydroxyl radical). These radicals are potent oxidizing agents that can quickly react with bacterial biomolecules, such as thiol groups of proteins, causing irreversible structural modifications and subsequent cellular death [96]. HP represents one of the most used biocides for different antimicrobial applications, such as disinfection and sterilization, and is colorless and odorless, and associated with low ecotoxicity. It is a versatile disinfectant, due to the possible employ in several environments including air, water, and surfaces. [97]

The most employed formulations of hydrogen peroxide are liquid and gas. Hydrogen peroxide liquid formulations are widely used for sterilization and disinfection processes. Usually, a 6% aqueous solution of hydrogen peroxide is employed for laboratory surface cleaning, but its bactericidal and sporicidal efficacy is lower against resistant bacterial spores and protozoan cysts, because of the short exposure time [98]. Hydrogen peroxide solutions are unstable thus suitable stabilizing agents such as benzoic acid are usually added. On the other hand, the production of non-toxic and biodegradable decomposition products (oxygen and water) emerges as an important advantage compared to other disinfectants [99].

Many studies revealed the effectiveness of the vaporized form of HP (HPV) for surface disinfection [100]. This system inactivates nonenveloped viruses, mycobacteria, and some multidrug-resistant microorganisms present in hospital room surfaces, reducing the number of contaminated porous and nonporous surfaces to 5–0% [101]. In particular, HPV resulted to be efficient against enteric and respiratory pathogens, including adenovirus type 5, poliovirus Sabin 1, rotavirus SA11, but also *Mycobacterium tuberculosis* and *C. difficile* spores [102]. In addition, HPV is often found in combination with heavy metals like silver ions, which showed an interesting bactericidal activity, resulting as a useful agent for surface disinfection in hospital settings [95,97]. The hydrogen peroxide solution in nebulization systems was also evaluated for surface disinfection. It provides a better decrease of microbial contamination on vertical surfaces compared to horizontal ones. However, the use of aerosol form is limited to the hospital’s empty spaces, excluding patient rooms, intensive care units, and other occupied areas [103]. 

Peracetic acid (CH_3_COOOH), Figure 13, is an organic peroxide with activity against mycobacteria, viruses, spores, molds at low concentrations. It results to be a more potent antimicrobial agent than hydrogen peroxide [104,105]. Peracetic acid is a strong oxidizing agent that provides innocuous decompositions by-products: acetic acid and hydrogen peroxide. Generally, it is employed as a surface disinfectant and for medical device sterilization [106]. A 15% aqueous solution of a mixture of peracetic acid, acetic acid, hydrogen peroxide, and water is commonly commercially available for the application as a disinfectant [99].

Figure 13 reports also performic acid (CH_2_O_3_), which is another well-known disinfectant characterized by virucidal, bactericidal, sporicidal, and fungicidal activity, useful in hospital environments and the food industry [107]. In a similar way to peracetic acid, performic acid liquid formulation includes formic acid, hydrogen peroxide, and water, with the production of non-toxic by-products. The main limit of performic acid solution application is due to its instability, which requires instant preparation before use [108].

### 4.5. Ozone 

Ozone (O_3_) is an inorganic gas, an allotropic form of oxygen, that represents one of the most potent oxidizing agents, mainly used for the disinfection of water systems but also for the decontamination of surfaces in healthcare settings and medical industries [109,110,111]. Ozone effectively inactivates bacteria, viruses, molds, and protozoa by producing hydroxyl free radicals that can react with glycoproteins; disrupting the integrity of the cell membrane; oxidizing enzyme’s thiol groups thus interfering with their activity; damaging DNA [112]. *P. fluorescens*, *S. aureus*, enteropathogenic *E. coli*, *S. typhimurium*, stomatitis virus, encephalomyocarditis virus, *Vibrio cholerae*, and *Shigella flexneri* are among the most sensitive microorganisms to the ozone treatment. Moreover, a quicker inactivation is observed when they are suspended in phosphate-buffered saline solutions [113]. 

O_3_ spontaneously decomposes into oxygen (O_2_) and a single reactive oxygen atom, associated with antimicrobial activity. On the other hand, the use of the gaseous form for disinfection is not convenient for operator safety, due to the exposure time to high concentrations of the gas [114]. Ozone solutions in water (ozonated water) allow one to obtain a liquid form useful for safe and effective surface disinfection, even if its low stability limited the applications [112]. In fact, the aqueous form shows a short half-life at 20 °C, approximately 20–30 min, after which it converts into an oxygen molecule; while the gaseous form results in having more stability and a longer half-life (12 h) [115]. The main aspects that affect ozone stability are temperature, pH, and ozone-oxidizable materials. To reduce the decomposition rate of the gas, several ozone generators were designed to produce a stabilized form of aqueous O_3_ and to extend its half-life up to a few hours [114]. 

The effectiveness of aqueous and gaseous O_3_ against manure-based pathogens (MBP) were assessed for several contaminated surfaces. Aqueous ozone achieved a good reduction of MBP contamination on plastic and metal surfaces after 4 min of exposure, but not in more complex surfaces [112]. In a recent study, aqueous ozone demonstrated its efficacy also against several isolates of SARS-CoV-2 after 5 min of incubation, resulting in a new potential alternative for the disinfection of outdoor surfaces contaminated by this virus [114,116]. Synergistic effects have been shown between ozone and ultraviolet, hydrogen peroxide, or negative air ions, to increase the production of hydroxyl radicals and to improve the antimicrobial activity [117]. Zoutman et al. evaluated the efficacy of ozone in combination with hydrogen peroxide in vapor form for steel surface disinfection, demonstrating a high level of decontamination in short exposure time against the most common hospital-associated microorganisms [118]. The combination of O_3_ at low concentration and ultraviolet also demonstrated synergistic effects on *E. coli* and *Escherichia* virus MS2 inactivation, highlighting the potential antimicrobial properties of this mixture couple for the development of new disinfectants [119]. The use of ozone generators may be associated with the production of negative air ions (NAI) and nitrogen oxides that displayed bacteriostatic properties and a reduction of microbial populations, alone and in combination with the O_3_ [120]. 

### 4.6. UV

Ultraviolet (UV) is electromagnetic radiation characterized by a wavelength from 10 to 400 nm, longer than X-rays but shorter than visible light. Three bands of UV light have been identified: UVA (400–315 nm), UVB (315–280 nm), and UVC (280–100 nm). UVC is also called ultraviolet germicidal irradiation (UVGI) for its antimicrobial properties [121]. In fact, for many years UV radiation has been employed for disinfection and sterilization, mainly the wavelength of 250 nm that has revealed better performance [122]. Nevertheless, different inactivation responses have been observed for several pathogens types including bacteria, viruses, fungi, and spores, even multidrug-resistant (MDR) strains of *Acinetobacter baumannii*, and *C. difficile* spores [123]. The efficacy of the decontamination is also related to the UVC amount and exposure time. For example, the best inactivation response for bacteria is at 254 nm, while higher wavelengths are required for viruses and protozoa (260–270 nm) [124]. 

The mechanisms involved in the antimicrobial effects of UV light are based on photochemistry. Microorganism biomolecules, mainly nucleotides, absorb the photon energy emitted by UV light which causes chemical modifications and cellular damage through three potential routes: photohydration of DNA, photosplitting (breaking the DNA), or photodimerization [121]. Usually, when thymine bases adjacent to other ones are excited by a UV light, several covalently linked dimers are generated, blocking the DNA replication process. Anyway, UV is not able to kill microorganisms but make them unable to duplicate and induce infections [125]. 

During the years the use of several UVC light-based devices for cleaning and disinfection especially in hospital settings is increased because of its associated advantages, among which the absence of residues after treatment, the broad-spectrum activity, and rapid exposure times [126]. Today, mercury vapor arc lamps and xenon lamps represent the most frequently used UVC devices (100–280 nm). The first one emits a continuous UVC light at low pressure (approximately 254 nm), while xenon lamps generate a pulsed light at high intensity [127]. However, UV irradiation at 254 nm can cause eyes and skin damages, so the treatment must be performed in unoccupied rooms. Alternatively, 222 nm UVC light could be used as it is poorly absorbed by the eyes and skin. Hiroki Kitagawa et al. validated the effectiveness of UVC radiation at 222 nm against SARS-CoV-2 contaminations, highlighting the possibility to carry out the disinfection process also in occupied rooms and spaces [128]. 

New technologies have been reported with the aim to improve the effectiveness of surface decontamination using UVGI. A novel portable UVC device has been assessed on several surfaces including plastic, bedrail, stainless steel, chrome-plated, and porcelain objects. A high level of bacterial inactivation has been observed against MRSA on the bedrail and against VRE on chrome and stainless steel [129]. Another study has described the efficacy of a new portable pulsed ultraviolet (UV) radiation generator for surface cleaning, towards the most common nosocomial bacteria, including *P. aeruginosa, A. baumannii*, *S. aureus*, and *B. cereus*. A potent antibacterial activity has been detected after a short exposure time, revealing an advantageous new method of sanitation [130,131]. Moreover, the UV technology leads to the development of the UVC reflective wall, aimed to reduce the time of irradiation. The exposure time decreases from 25 to 5 min for MRSA and 43 to 9 min for *C. difficile* spores if a UVC generator (254 nm) is located in a room coated by a specific reflective agent for UVC light [132]. 

The different mechanisms of action, the antimicrobial and cellular effects of the described antimicrobial agents are summarized in Table 5 together with the main advantages and disadvantages. 

## 5. Antimicrobial Surfaces 

To date, several strategies have been proposed to prevent microorganisms from adhering to the surface or to kill the ones that manage to attach them. Furthermore, minimizing biofilm formation should be a further goal [133]. Nonetheless, it is necessary to take into account that bacterial colonization of surfaces is a key process of corrosion, infection, fermentation, and fouling [134].

**Figure 14:**
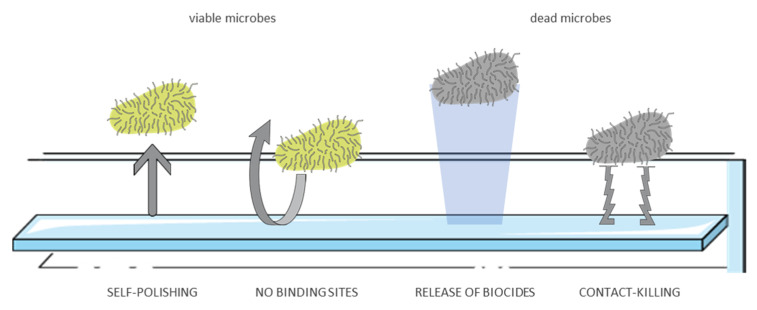
General classes of antimicrobial surfaces.

New strategies to control and hopefully avoid the adhesion of microorganisms on surfaces (Figure 14) are inspired by nature, a source that appears to be almost unlimited, and it has attracted a large amount of interest in the past decades. Indeed a current trend is based on natural materials such as plant leaves and insect cuticles. For example, the leaves of *Nelumbo nucifera*, commonly known as lotus, exhibit superhydrophobicity, and self-cleaning abilities. The characteristics that afford this self-cleaning capability are the lipid layer that covered the surface. This results in a high water contact angle (θ > 150°) and a low tilting angle (θ < 10°), which are parameters needed to lead the water droplet to roll off [135]. In this way, the water droplets collect dirt as they move over the leaf. Many other plants exhibit very similar properties to that of the lotus leaf, Indian canna, taro, and cabbage leaves.

Similarly, insect surfaces are covered by a layer of lipophilic cuticle. Some insects, e.g., dragonflies or cicada, self-assemble this barrier into three-dimensional nanoarray structures, which enable air to be trapped in and hence exhibit a high water contact angle [136,137]. Furthermore, the turbulent conditions during their flight enhance these self-cleaning properties. Artificial surfaces can be produced to possess similar properties, causing water to behave in a similar way, therefore bacterial cells could be removed before they could adhere to the surface [134,138].

Other interesting approaches use bio-functionalization or surface coatings to give or enhance antibacterial properties: solid heavy metals, such as silver [139,140], copper [141,142,143] or zinc [144,145], and its alloys have been widely used as antimicrobial agents for millennia due to their intrinsically strong antibacterial activity. 

Usually, these approaches focus on a nano-size particulate form of the metal: a larger surface allows better contact with the target microbe cells while enabling a more efficient release of the particles. Among these materials, copper is one of the most frequently used due to its efficiency in “contact killing”: microorganisms survive only a few minutes on these kinds of surfaces [146,147]. Obviously, the higher the copper concentration, the faster and more efficient is the antimicrobial activity. Nevertheless, to promote the activity other factors have to be taken into account: both extrinsic, such as protocols and operators, and intrinsic [148]. 

The major issue with the use of metallic ions is that their interactions are non-specific, which is a major concern from a biocompatibility and cytotoxicity point of view. Furthermore, the leaching components may contaminate and accumulate in the environment, promoting bacteria’s resistance.

Further studies are still required to find the best enhancing parameters like high temperature or high humidity, the metal’s physical form, or coating techniques [149].

More recently, another innovative approach based on photosensitizer compounds has been developed for preventing bacterial colonization. These biocides exert their action after activation by a light source [150]. UVA-induced antimicrobial activity can also be achieved with metals [151,152]; the main mechanisms driving the activity are the formation of highly reactive species like superoxide and hydroxyl radicals and the slow release of metal ions.

The most common techniques that can be applied to incorporate biocides in the surface involve the impregnation of the antimicrobial into the coating. The simultaneous encapsulation of different antimicrobials in one matrix has proven to be more efficient than entrap only one [153]. The layer by layer (LbL) technique is another powerful strategy for surface engineering, which allows one to control the leaching characteristics of a biocide [154].

**Figure 15:**
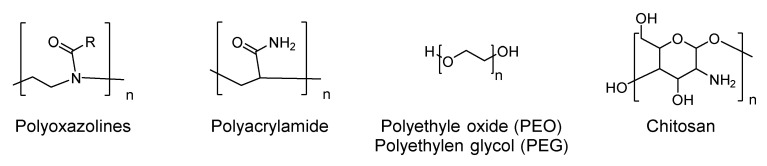
Chemical structure of some common monomers and polymers used for surface treatment.

In addition, slow-releasing systems, release-on-command systems, and non-leaching systems have also been developed. Commonly employed polymers are polyoxazolines with methyl (PMOZ), ethyl (PEOZ), and propyl (PPOZ) [155], polyacrylamide [156], or poly ethylene glycol-PEG [157] ). It has been experimentally proven that antimicrobial properties are also shown by surfactant type polymers and some naturally derived polymers, like chitosan [158]. Different molecules used to chemically modify the surface are described in Figure 15. The building blocks of these polymers can differ in nature, molecular weight, and chain length. These are critical parameters that need to be optimized with other factors which may influence the effectiveness of the antimicrobial, like the surface charge density and the hydrophilic/hydrophobic balance. 

The physical principle is that polymer brushes act as a steric barrier against bacterial attachment. Indeed some polymers provide an unfavorable surface for bacterial interaction, especially cationic polymers. They have shown effectiveness against bacterial infection but their long-term use discloses toxicity as a concern. Their mechanism totally relies on their charge that attracts and “captures” negatively charged bacterial cells, and this interaction damages the bacterial membrane, giving a bacteriostatic, and eventually a bactericidal effect.

To improve the antimicrobial efficacy several agents, such as small compounds, peptides, and enzymes, can be introduced into polymer molecules [159]. Probably, polymers of QACs represent the class that has received more attention over the years [160,161].

Ideally, a coating of the antimicrobial polymer must exhibit a broad antimicrobial spectrum in a brief contact time and it must remain effective over the lifespan’s article while avoiding leaching into the environment or decomposition in toxic products. Furthermore, it shouldn’t be toxic nor irritating to those who are handling it and not water-soluble (for water disinfection application) [162].

**Figure 16:**
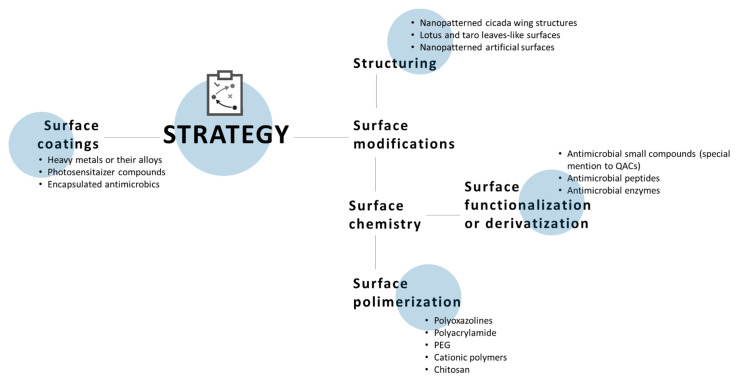
Different approaches in the design of antimicrobial surfaces.

Figure 16 summarizes all the approaches that involve changes in the chemical and/or physical properties of the surface to have a biocide effect.

## 6. Current and Future Issues

Antimicrobials are a precious resource that effectively keeps harmful microorganisms at bay. Unfortunately, nowadays, biocidal products are perceived as either direct and indirect threats. The direct one is due to the dissemination of resistant strains: the concept of bacterial resistance to biocides is not novel and the first evidence has been reported in the early 1950s [163]. This phenomenon has been associated with the increasing exposure to biocides; furthermore, several investigations describe a possible linkage between antimicrobial agents and the occurrence of antibiotic cross- and co-resistance [164,165]. The indirect threat regards the transfer of genes which confers resistance to a susceptible strain, enhancing its resistance level. Among the several routes for the transfer of genes, horizontal gene transfer (HGT) [166,167] enables the exchange of transposons, integrons, and plasmids where antibiotic resistance genes are generally located. Lu et al. [168] demonstrate that high environmental concentrations of triclosan promote HGT of multidrug resistance genes between bacteria providing resistant strains. Another example regards the extensive use of QAC that has been blamed for the spread of QAC-resistance bacteria, both Gram-positive and negative. Resistance’s mechanisms to this class of compounds are underexplored, however, efflux pump and alteration of membrane composition are among the predominant ones [169,170]. 

Another example of antimicrobial resistance can be found in the tolerance to oxidizing biocides, like chlorine, hydrogen peroxide, and peracetic acid, which has also been described [171]. Resistance to these agents can result from the overproduction of enzymes which increases the defense towards radical-mediated damage or protects from biofilm’s alterations. 

The selective pressure towards disinfectants may occur also when biocides are discharged into the environment, themselves, or their residues [172,173]. McBain et al. [174] investigate the effects of triclosan use on the domestic-drain biofilm ecosystems. They found out that the biocide did not significantly lower the total counts but altered the bacterial composition, due to innate resistance or insusceptibility of some species able to degrade triclosan. Hospital wastewaters have been investigated as well [175,176] as they are characterized by a high concentration of antibiotics and disinfectants.

However, the lack of data on the majority of antimicrobial compounds prevents one from clearly identifying the risk arising from the increase and indiscriminate use of these biocides.

In conclusion, the consciousness that the perfect antimicrobial agent may not yet exist the right choice and the appropriate use of the current chemicals are necessary to avoid both resistance and environmental issues. For this purpose, a deep knowledge of the antimicrobial agent together with the type of surface would result in an effective and suitable disinfection level.

## Figures and Tables

**Table 1:** Common endospore-producing bacteria and their clinical manifestations.

Bacterial Species	Clinical Manifestation
*B. anthracis*	anthrax
*B. cereus*	foodborne illness
*B. subtilis*	not pathogen
*C. botulinum*	botulism
*C. perfringens*	gas gangrene
*C. tetani*	tetanus

**Table 2:** Classification of biological agents.

Risk Classification	Description	Examples	Heading
Category 1	Pathogen with a low probability of developing diseases in the human organism	Nonpathogenic strains of *Escherichia*	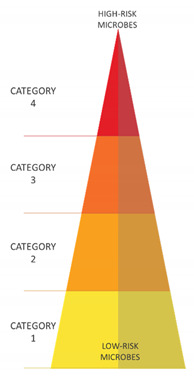
Category 2	Pathogen that may cause pathology in humans and be a potential hazard for workers; it’s unlikely that can be spread in the community; usually, there are effective treatments	Measles virus, *Salmonella, Legionella*	
Category 3	Pathogen that may cause severe illness in humans and be a serious hazard for workers; the biological agent may spread in the community, but usually effective treatments are available	HIV, *Bacillus anthracis*, HBV, HCV, *Mycobacterium tuberculosis* SARS-CoV-2	
Category 4	Pathogen that may cause severe illness in humans and may be a serious hazard for workers; the biological agent can spread in the community, and usually, there are no effective treatments available. Pathogens with a low probability of developing diseases in human organisms. Pathogens that may cause pathology in humans and be a potential hazard for workers; it is unlikely that they can be spread in the community; usually, there are effective treatments	Ebola virus, Lassa virus, Smallpox virus. Nonpathogenic strains of *Escherichia* Measles virus, *Salmonella, Legionella*	

**Table 3:** Effects of pH level on antimicrobial activity.

Activity as Environmental pH Increases	Classes of Disinfectants	Mechanisms
Decreased activity	Phenols and organic acids	Increase in the degree of dissociation of the molecules
	Hypochlorites	Undissociated hypochlorous acid is the most fast-acting species
	Iodine	At low pH, iodine, the most powerful antimicrobial species, is the dominating one
Increased activity	Quaternary ammonium compounds (QACs)	Increase in the degree of ionization of bacterial surface groups leading to an increase in binding

**Table 4:** Recommended dilutions of commonly used chlorine releasing compounds.

Chlorine Type	Use Condition		Advantages	Disadvantages
	Clean Condition	Dirty Condition		
Sodium hypochlorite solution (5% available chlorine)	20 mL/L	100 mL/L	Can be local (stabilized form) Can be on-side (no stabilized form Does not clog pipes	Shorter shelf life Difficult to ship Low stability (no stabilized form)
High-test hypochlorite (70% available chlorine)	1.4 g/L	7.0 g/L	Easy to ship Long shelf life	Explosive
Sodium dichloroisocyanurate powder (60% available chlorine)	1.7 g/L	8.5 g/L	Easy to ship Long shelf life Does not clog pipes	Smell
Sodium dichloroisocyanurate tablets (1.5 g available chlorine per tablet)	1 tablet per L	4 tablets per L	Easy to ship Long shelf life Does not clog pipes	Smell

**Table 5:** Summary of advantages and disadvantages of common surface disinfectant.

Disinfectant	Mechanism of Action	Cellular Effect	Antimicrobial Effect	Advantages	Disadvantages
Chlorine compounds	Oxidation of side chains amino acids in proteins	Unfolding tertiary structure and protein aggregation	Bactericidal, fungicidal, virucidal sporicidal	-Not flammable -Fast-acting -Low-cost -Resistant to water hardness -Relatively stable	-Salt residues -Corrosive to metals -Affected by organic matter -Fabric discoloration -Potential production of trihalomethane -Irritating odor at high concentrations
Iodine compounds	Oxidation of thiol groups to disulfides in proteins	Modification of structural protein and/or alterations in enzyme activities	Bactericidal, virucidal	-Not flammable	-Limited spectrum of activity -Degradation of silicone catheters -Staining for surfaces
Alcohols	Denaturation and precipitations of cytoplasmic and membrane proteins	Alteration in metabolic processes, membrane damage	Bactericidal, fungicidal, virucidal	-Fast-acting -Noncorrosive -Nonstaining -Suitable for small surfaces disinfection	-Not sporicidal -Affected by organic matter -No cleaning properties -Deterioration of some instruments -Flammable -Rapid evaporation
Phenols	Denaturation of cytoplasmic and membrane proteins	Leakage of essential metabolites, release of K^+^, membrane damage, cytoplasmic coagulation	Bactericidal, fungicidal, virucidal	-Low costs -Not flammable -Nonstaining	-Rapid absorption by porous materials and irritate tissues -Potential depigmentation of skin -Hyperbilirubinemia in infants
Quaternary ammonium compounds	Binding to phosphates and fatty acid chains in phospholipids of cell membrane and DNA	Depolarization, membrane damage, cytoplasmic coagulation	Bactericidal, fungicidal, virucidal (enveloped viruses)	-Good cleaning agents -Surface compatible -Long antimicrobial activity -Low costs	-Not sporicidal -Affected by water hardness -Asthma after benzalkonium chloride exposure -Affected by organic matter
Hydrogen peroxide and peracids	Oxidation of thiol groups to disulfides in proteins	Modification of structural protein and/or alterations in enzyme activities	Bactericidal, fungicidal, virucidal	-Fast-acting -Safe for workers -Non-toxic by-products -Surface compatible -Nonstaining -Odorless -Not flammable	-More expensive compared to other disinfectants -Not sporicidal at low concentrations
Ozone	Oxidation of thiol groups in proteins and interaction with purine and pyrimidine bases	Modification of structural protein, alterations in enzyme activities, and/or DNA damages	Bactericidal, moldicidal, virucidal, protozocidal	-Fast-acting	-Gaseous form not safe -Low stability solutions form -Reacted with organic matter
UV light	chemical modifications of nucleotides caused by photon energy emitted	DNA damages (photohydration, photosplitting, photodimerization)	bacteria, fungi, viruses, spores	-Absence of residues or by-products -Fast-acting	-No microbiocidal effect -Eyes and skin damages for UV irradiation at 254-nm

## Data Availability

Not Applicable.
